# Platelets Image Classification Through Data Augmentation: A Comparative Study of Traditional Imaging Augmentation and GAN-Based Synthetic Data Generation Techniques Using CNNs

**DOI:** 10.3390/jimaging11060183

**Published:** 2025-06-04

**Authors:** Itunuoluwa Abidoye, Frances Ikeji, Charlie A. Coupland, Simon D. J. Calaminus, Nick Sander, Eva Sousa

**Affiliations:** 1 Centre of Excellence for Data Science, Artificial Intelligence and Modelling, University of Hull, Hull HU6 7RX, UK; i.abidoye-2021@hull.ac.uk (I.A.); ikejif@gmail.com (F.I.); 2 Biomedical Institute for Multimorbidity, Centre for Biomedicine, Hull York Medical School, University of Hull, Hull HU6 7RX, UK; 3 Tut-All Software GmbH, 76275 Ettlingen, Germany

**Keywords:** platelets, data augmentation, GAN, WGAN-GP, CNN, transfer learning, medical imaging

## Abstract

Platelets play a crucial role in diagnosing and detecting various diseases, influencing the progression of conditions and guiding treatment options. Accurate identification and classification of platelets are essential for these purposes. The present study aims to create a synthetic database of platelet images using Generative Adversarial Networks (GANs) and validate its effectiveness by comparing it with datasets of increasing sizes generated through traditional augmentation techniques. Starting from an initial dataset of 71 platelet images, the dataset was expanded to 141 images (Level 1) using random oversampling and basic transformations and further to 1463 images (Level 2) through extensive augmentation (rotation, shear, zoom). Additionally, a synthetic dataset of 300 images was generated using a Wasserstein GAN with Gradient Penalty (WGAN-GP). Eight pre-trained deep learning models (DenseNet121, DenseNet169, DenseNet201, VGG16, VGG19, InceptionV3, InceptionResNetV2, and AlexNet) and two custom CNNs were evaluated across these datasets. Performance was measured using accuracy, precision, recall, and F1-score. On the extensively augmented dataset (Level 2), InceptionV3 and InceptionResNetV2 reached 99% accuracy and 99% precision/recall/F1-score, while DenseNet201 closely followed, with 98% accuracy, precision, recall and F1-score. GAN-augmented data further improved DenseNet’s performance, demonstrating the potential of GAN-generated images in enhancing platelet classification, especially where data are limited. These findings highlight the benefits of combining traditional and GAN-based augmentation techniques to improve classification performance in medical imaging tasks.

## 1. Introduction

Platelets, as essential cellular components of blood, play a critical role in hemostasis by preventing excessive bleeding at injury sites and maintaining vascular integrity [1,2,3]. These small, anucleate cells, also known as thrombocytes (2–4 μm in diameter), form aggregates to halt bleeding by adhering to damaged vessel surfaces, becoming activated, and transforming into sticky plugs that culminate in thrombus formation [4,5]. Beyond their mechanical role, platelets are regulated by intricate biochemical pathways, such as those involving endothelial cells that produce prostacyclin (PGI_2_) to modulate cyclic adenosine monophosphate (cAMP) levels via adenylyl cyclase (AC) [5]. Zinc further influences this signaling by altering AC and phosphodiesterase activities, where deficiencies can lead to excessive cAMP production, impaired clotting, and increased bleeding risk [6]. While red and white blood cells often receive more attention due to their ease of analysis, precise identification of platelet morphology and function is vital for diagnosing hematological disorders, as irregularities in shape or behavior may signal defective function and underlying pathologies [7,8,9,10].

Traditional platelet classification relies on manual microscopic examination, a labor-intensive process prone to inter-observer variability and subjectivity [2,11,12]. This has spurred interest in automating blood analysis to enhance efficiency and reduce human error [13,14]. Artificial Intelligence (AI), particularly Machine Learning (ML) and Deep Learning (DL), is revolutionizing biomedical research and diagnostics by enabling rapid, accurate analysis of microscopy images [15,16]. DL techniques, such as Convolutional Neural Networks (CNNs), have shown remarkable success in medical imaging tasks like tumor detection, blood cell classification, and phenotyping, offering high-performance solutions for classification, segmentation, and image quality enhancement [17,18,19,20]. For platelets, CNNs have been applied to classify aggregates by agonist type and to phenotype morphological changes induced by treatments, demonstrating their potential to automate and refine analysis [2,5,21].

Despite these advancements, a key challenge persists: the scarcity of high-quality, labelled platelet image datasets [22]. Traditional data augmentation techniques, such as rotations, scaling, and flips, provide some relief [23], but Generative Adversarial Networks (GANs) have emerged as a transformative approach by synthesizing realistic images to expand datasets [24]. GAN-based augmentation has improved classification accuracy in applications like liver lesion detection and chest X-ray analysis, suggesting its potential for platelet studies [25,26]. Building on this, recent research has explored custom CNN architectures and transfer learning to classify blood cells, including platelets, with models like PlateNet designed to identify morphological changes induced by treatments such as zinc, milrinone, or their combination [5,21]. PlateNet, for instance, leverages annotated datasets to classify platelet groups, offering insights into how signaling modulation affects function, with implications for understanding platelet disorders [5].

To address dataset limitations and advance platelet classification, this study compared a Wasserstein GAN with Gradient Penalty (WGAN-GP)-generated dataset against traditionally augmented datasets. The performance of eight pre-trained CNNs (DenseNet121, DenseNet169, DenseNet201, VGG16, VGG19, InceptionV3, InceptionResNetV2, and AlexNet) and two custom CNNs was evaluated using accuracy, precision, recall, and F1-score across original, augmented, and GAN-based synthetic datasets. The objective was to determine whether GAN augmentation enhances classification accuracy and generalizability beyond traditional methods while also exploring AI’s broader potential to decode platelet morphology and signaling in health and disease.

## 2. Materials and Methods

### 2.1. Data Description

The initial dataset comprises 71 platelet images, categorized into three classes, Control (47 images), Milrinone (14 images), and Zinc plus Milrinone (10 images), as described by Abidoye et al. [27]. These images were sourced from a study by Coupland et al. [5], where platelets were obtained from blood samples donated by consenting adults under ethics authorization by the Hull York Medical School Ethics Committee for “The study of platelet activation, signaling, and metabolism” and the National Health Service (NHS) Research Ethics Committee (REC) study “Investigation of blood cells for research into cardiovascular disease” (21/SC/0215). The images were captured using high-resolution microscopy, focusing on platelet morphology under different treatment conditions to study signaling pathways and thrombus formation.

**Figure 1:**
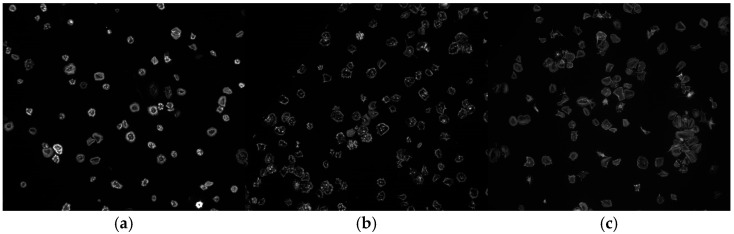
Sample images from the platelet dataset of each class: (**a**) Control, (**b**) Milrinone, (**c**) Zinc plus Milrinone, respectively.

The platelet images are grayscale microscopy images stored in JPEG format. Each image used has a resolution of 128 × 128 pixels with an 8-bit pixel intensity depth, providing 256 levels of gray. These properties ensure compatibility with the CNN architectures used in this study. An example of the platelet images can be seen in Figure 1.

This small dataset size presented challenges for training deep neural networks, motivating our augmentation strategies.

### 2.2. Data Preparation and Organization

To address the class imbalance in the original dataset (47 Control, 14 Milrinone, 10 Zinc plus Milrinone), random oversampling was applied in Level 1 augmentation to balance classes, resulting in 47 images per class (total 141 images). In Level 2 augmentation, extensive transformations were applied to further increase the dataset size, maintaining approximately equal distribution, with 488 Control, 487 Milrinone, and 488 Zinc plus Milrinone images (total 1463 images). This ensured balanced representation across classes, reducing bias in model training. Each dataset was then divided into training (70%) and validation (30%) sets.

### 2.3. Transfer Learning Models

Eight pre-trained CNNs with ImageNet weights were fine-tuned for this task:

- DenseNet121, DenseNet169, DenseNet201 [28]
- VGG16, VGG19 [29]
- InceptionV3 [30]
- InceptionResNetV2 [30]
- AlexNet [31]

Each model’s final layers were adjusted for three-class classification. Additionally, two custom CNNs were designed:

- Custom Model 1: Incorporating Conv2D, BatchNormalization, MaxPooling, Dropout, and two Dense layers.
- Custom Model 2: A simpler architecture with Conv2D and MaxPooling, followed by a single Dense layer.

### 2.4. Data Augmentation (GAN Data Generation)

**Figure 2:**
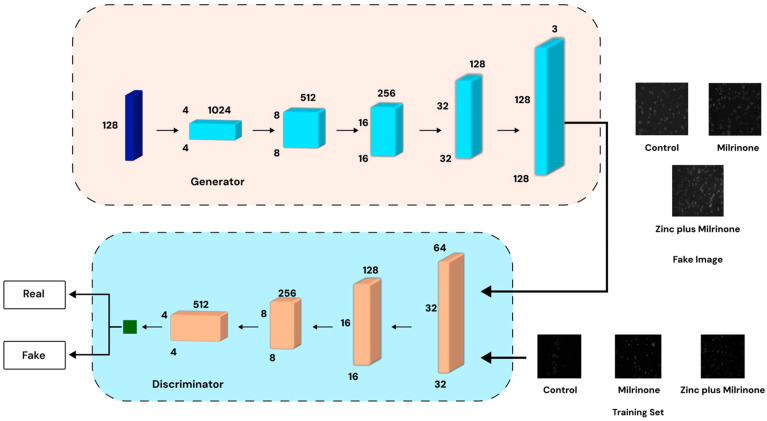
WGAN-GP architecture for synthetic platelet image generation. The generator (**top**) produces synthetic images, while the discriminator (**bottom**) evaluates real vs. generated images to refine quality.

A Wasserstein GAN with Gradient Penalty (WGAN-GP), as illustrated in Figure 2, was employed to generate synthetic platelet images. This method was chosen due to its ability to address stability issues commonly encountered in traditional GAN training by utilizing the Wasserstein distance with a gradient penalty to enforce Lipschitz continuity [32,33].

1. Generator: This network transforms a latent noise vector into high-resolution (128 × 128 or 256 × 256 pixels) synthetic images using transpose convolutions, batch normalization, and LeakyReLU activations to ensure realistic feature generation.
2. Critic (Discriminator): The discriminator evaluates both real and generated images through convolutional layers, outputting a scalar “realness” score to guide the generator’s improvement. To maintain training stability, the critic undergoes multiple updates per generator update before reaching convergence.

GAN training was conducted over 5000 epochs per class (batch size = 128), generating 100 synthetic images per class. The synthetic dataset of 300 images (100 images per class: Control, Milrinone, Zinc plus Milrinone) was used as an independent dataset, separate from the Level 1 and Level 2 augmented datasets, to evaluate the effectiveness of GAN-generated images in isolation. These images were subjected to the same CNN training and evaluation pipeline for direct comparison with other datasets.

### 2.5. Model Training and Evaluation

#### 2.5.1. Model Training

To ensure unbiased evaluation, each dataset was split into training (60%), validation (20%), and test (20%) sets. The test set was reserved for final model evaluation after hyperparameter tuning and training, ensuring performance assessment on unseen data. For the original dataset (71 images), this resulted in 43 training, 14 validation, and 14 test images. For Level 1 (141 images), the split was 85 training, 28 validation, and 28 test images. For Level 2 (1463 images), it was 878 training, 293 validation, and 292 test images. For the GAN-augmented dataset (300 images), it was 180 training, 60 validation, and 60 test images, and all CNN models were trained for 100 epochs using the following:

- Optimizer: Adam with a learning rate of 0.001.
- Batch Size: 32 (128 tested in some trials).
- Loss Function: Categorical cross-entropy for the multi-class classification.

Hyperparameters were tuned for optimal performance. The best-performing checkpoints were saved.

#### 2.5.2. Evaluation Metrics

The evaluation metrics used were as follows:

(1) $$Precision=\frac{TP}{TP+FP}$$

(2) $$Recall=\frac{TP}{TP+FN}$$

(3) $$Accuracy=\frac{TP+TN}{TP+TN+FP+FN}$$

(4) $$F1Score=2*\frac{\left(Precision*Recall\right)}{\left(Precision+Recall\right)}$$

Confusion matrices, accuracy, and loss plots were generated to visualize classification performance.

## 3. Results

### 3.1. Original Dataset (71 Images)

As shown in Table 1, the limited size of the original dataset resulted in moderate model performance. DenseNet121 achieved the highest accuracy at 81%, with a precision of 84%, while other architectures like DenseNet201 and DenseNet169 followed, with 76% and 71% accuracy, respectively. However, VGG19 struggled, attaining only 52% accuracy, and the two custom CNN models underperformed significantly, particularly Custom Model 2, which had a precision of only 33%. These results highlight the challenge of training deep learning models with small datasets, reinforcing the need for data augmentation to improve generalizability.

To evaluate whether models trained on augmented datasets improve classification on non-augmented data, the best-performing models (InceptionV3, InceptionResNetV2, DenseNet201) from Level 2 and GAN-augmented datasets were tested on the original dataset’s test set (14 images). InceptionV3 achieved 85% accuracy, DenseNet201 reached 82%, and InceptionResNetV2 scored 84% compared to 81% for DenseNet121 trained on the original dataset. These results suggest that augmentation enhances generalizability to real, non-augmented platelet images.

Note: The following tables report test set performance after splitting each dataset into training (60%), validation (20%), and test (20%) sets. Exact metrics were recomputed to reflect performance on unseen data, ensuring robust evaluation.

**Table 1:** Original dataset (71 images) model results.

Models (Batch Size 32)	Accuracy (%)	F1-Score (%)	Precision (%)	Recall (%)
Custom Model 1	62	56	68	62
Custom Model 2	57	42	33	57
DenseNet121	81	79	84	81
DenseNet169	71	67	77	71
DenseNet201	76	74	83	76
VGG16	57	47	43	57
VGG19	52	45	40	52
VGG19-FF	62	59	63	62
InceptionV3	62	51	46	62
InceptionResNetV2	71	69	76	71
AlexNet	62	56	59	62

### 3.2. Augmented Dataset Level 1 (141 Images)

As presented in Table 2, performance improved across most architectures compared to the original dataset. DenseNet201 achieved the highest accuracy at 86%, followed by DenseNet121, DenseNet169, and InceptionV3, which ranged between 79% and 76%. The application of augmentation techniques contributed to enhanced model generalizability. However, Custom Model 2 exhibited significantly lower performance, with an accuracy of only 38% and a precision of 15%, indicating its limited capacity to learn from the expanded dataset.

### 3.3. Augmented Dataset Level 2 (1463 Images)

A substantial jump in performance was observed with the dataset augmented to the Level 2, as shown in Table 3. In these experiments, InceptionV3 and InceptionResNetV2 reached 99% accuracy, with equally high precision and recall, underscoring the importance of a sufficiently large, varied dataset. DenseNet201 also performed exceptionally (98% accuracy).

### 3.4. GAN-Augmented Dataset (300 Images)

GAN-based augmentation further enhanced classification outcomes, as shown in Table 4. DenseNet121 and Custom Model 1 both achieved 97% accuracy, while Inception-based models and DenseNet169 also scored above 90%. Although AlexNet improved slightly (74% accuracy), it still lagged in comparison to more modern architectures.

The GAN-augmented dataset (300 images) was evaluated independently to assess the performance of models trained solely on synthetic images, providing insights into the quality and utility of WGAN-GP-generated data.

### 3.5. Synthetic Dataset Result

**Figure 3:**
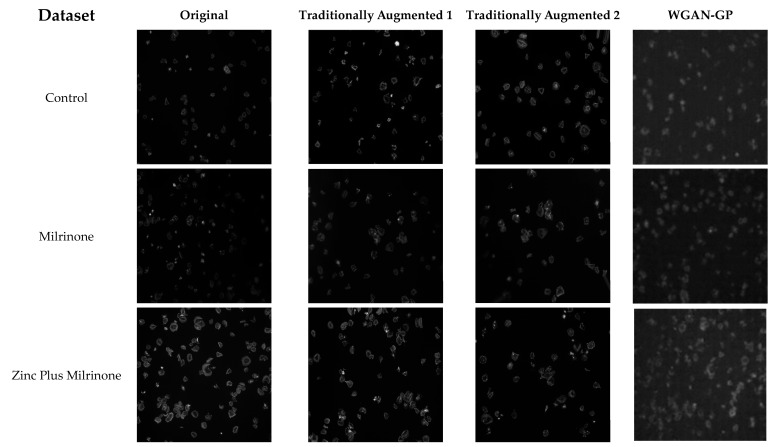
Comparison of original, traditionally augmented, and GAN-generated platelet images. Columns from left to right: Original dataset, Level 1 augmentation (basic transformations: flipping, rotation), Level 2 augmentation (extensive transformations: shearing, zooming), and WGAN-GP-generated synthetic images. Rows represent classes: Control, Milrinone, and Zinc plus Milrinone.

Figure 3 illustrates the differences between the original dataset and the various augmentation strategies applied to platelet images. The first column represents the original dataset, followed by the traditionally augmented Level 1 dataset, which applies basic transformations such as flipping and rotation. The third column showcases the traditionally augmented Level 2 dataset, incorporating more extensive modifications such as shearing and zooming. Finally, the last column presents images generated using a Wasserstein GAN with Gradient Penalty (WGAN-GP), producing synthetic platelet images that expand the dataset. The progressive transformations highlight the role of each augmentation technique in enhancing dataset diversity and model robustness.

## 4. Discussion

The methodological choices in this study, particularly concerning data augmentation and model selection, significantly influenced the results obtained. The original dataset, consisting of only 71 platelet images, presented a considerable limitation for training deep learning models, leading to suboptimal performance due to overfitting. As observed in previous studies, deep learning models require large and diverse datasets to generalize effectively, and small sample sizes often lead to performance degradation [23,34]. In this study, models trained solely on the original dataset demonstrated moderate performance, with DenseNet121 achieving the highest accuracy at 81%, while simpler architectures such as VGG19 and AlexNet performed significantly worse, with 52% and 62% accuracy, respectively. These findings align with previous work in medical image classification, where small datasets have been shown to negatively impact deep learning models, particularly those with complex architectures [23,34].

To mitigate this issue, two levels of traditional augmentation were applied. The first level (141 images) employed basic transformations, such as flipping, rotation, and zooming, whereas the second level (1463 images) incorporated more extensive modifications, including shearing and additional rotation angles. As reported in Perez et al. [23], data augmentation has been widely acknowledged as a key strategy to mitigate overfitting and improve model robustness in deep learning applications. In line with these findings, the results of this study demonstrated that Level 2 augmentation significantly improved model performance, particularly for deeper architectures. Models such as InceptionV3 and InceptionResNetV2 achieved 99% accuracy, reinforcing existing literature, which suggests that large, diverse datasets enable deep networks to extract more representative features, ultimately leading to superior classification performance [32].

The improved classification accuracy achieved with Level 2 and GAN-augmented datasets has significant practical implications for clinical diagnostics. Automated platelet classification can enhance the efficiency of hematological analysis, reducing reliance on labor-intensive manual microscopy. For instance, accurate identification of platelet morphology under different treatments (e.g., Milrinone or Zinc plus Milrinone) could aid in diagnosing platelet-related disorders, and guide personalized treatment strategies. The use of GAN-generated images further addresses data scarcity, enabling robust model training in resource-limited settings, such as smaller clinical laboratories.

A key limitation of this study is the potential unrepresentativeness of the original dataset, which includes only 71 images from a single study [5]. While augmentation strategies significantly increased dataset size and diversity, they do not introduce new information beyond the original samples. If the original dataset does not fully capture the variability of platelet morphology across diverse populations or conditions, the models may struggle to generalize to broader clinical scenarios. Future studies should prioritize collecting a larger, more representative dataset to enhance the applicability of the proposed methods.

Further improvements were observed with GAN-based augmentation, where a WGAN-GP model generated 300 synthetic platelet images after being trained for 5000 epochs per class. As seen in Yi et al. [24] and Frid-Adar et al. [25], GAN-generated data have been shown to effectively enhance deep learning models, particularly in medical imaging applications where data availability is limited. The introduction of synthetic images further improved classification outcomes, with DenseNet121 and Custom Model 1 achieving 97% accuracy, demonstrating the effectiveness of synthetic data generation in complementing real datasets. However, it is important to note that, while GAN-based augmentation provided substantial improvements, certain challenges remain. Prior studies have reported that GANs can suffer from mode collapse, where the model generates highly similar images, leading to a lack of diversity in the dataset [24]. In this study, the use of WGAN-GP helped mitigate this issue by ensuring more stable training and realistic image generation.

The presence of artifacts in Level 1 and Level 2 augmented images, such as distortions from shearing or zooming, raises concerns about their impact on model training. While these transformations increased dataset diversity, they introduced visual differences from the original images, potentially leading the models to learn artifact-specific patterns. To mitigate this, the augmentation pipeline was designed to include a range of transformations while preserving core platelet morphology. The high performance of the models on the Level 2 dataset (e.g., 99% accuracy for InceptionV3) suggests that these artifacts did not significantly hinder learning of true features. However, future work should employ artifact-aware augmentation techniques, such as adaptive augmentation, to minimize distortions and ensure that models generalize effectively to real-world microscopy images.

Comparative analysis of augmentation techniques indicates that traditional augmentation provided a strong foundation for improving model generalizability, but further enhanced classification accuracy has been achieved with the GAN-based augmentation by introducing synthetic variations. The expectation was that augmentation would improve model robustness, particularly for deeper architectures, and the results support this hypothesis. However, the degree of improvement varied across the models. Notably, AlexNet’s accuracy declined significantly to 30% on Level 2 augmentation, suggesting that its limited capacity for complex feature extraction made it less effective when trained on a highly varied dataset. Conversely, DenseNet and the Inception-based models exhibited substantial performance gains, aligning with the findings in Sandfort et al. [22], which highlighted that deeper networks require larger and more diverse datasets to optimize feature extraction and classification.

These results further support the broader consensus in the literature that data augmentation plays a pivotal role in improving model generalizability, mitigating overfitting, and addressing dataset limitations in deep learning applications. As demonstrated in previous studies, including those on liver lesion classification, chest X-ray diagnostics, and histopathology image analysis, augmentation techniques have consistently led to performance improvements across various medical imaging tasks [22,23,25]. Future research should investigate conditional GANs and domain adaptation strategies to enhance data augmentation, improving the diversity and quality of training samples. Additionally, transformer-based architectures should be explored for classification after augmentation, as they have recently shown strong performance in complex image classification tasks. Additionally, expanding the dataset with real-world platelet images and incorporating more sophisticated augmentation strategies, such as adaptive augmentation and meta-learning approaches, may further enhance the generalizability and robustness of platelet classification models.

Future work will incorporate k-fold cross-validation on the original non-augmented dataset to further validate the robustness of models trained on augmented data. This approach will help confirm that the proposed augmentation strategies enhance performance on real-world platelet images without relying solely on synthetic or transformed data.

## 5. Conclusions

This study investigated platelet image classification using traditional data augmentation (Levels 1 and 2) and a WGAN-GP approach to generate synthetic data. The results showed that extensively augmented datasets (Level 2) and GAN-augmented data both significantly improved classification accuracy for advanced CNN architectures (particularly DenseNet and Inception families). These outcomes underscore the value of combining comprehensive augmentation strategies with GAN-based synthetic images, especially in cases where medical image data are limited.

## Figures and Tables

**Table 2:** Augmented dataset Level 1 (141 images) model results.

Models (Batch Size 32)	Accuracy (%)	F1-Score (%)	Precision (%)	Recall (%)
Custom Model 1	67	66	69	67
Custom Model 2	38	21	15	38
DenseNet121	79	79	79	79
DenseNet169	79	78	83	79
DenseNet201	86	86	88	86
VGG16	62	62	72	62
VGG19	64	64	68	64
VGG19-FF	76	76	80	76
InceptionV3	76	76	79	76
InceptionResNetV2	71	69	80	71
AlexNet	67	65	66	67

**Table 3:** Augmented dataset Level 2 (1463 images) model results.

Models (Batch Size 32)	Accuracy (%)	F1-Score (%)	Precision (%)	Recall (%)
Custom Model 1	97	97	97	97
Custom Model 2	88	87	91	88
DenseNet121	97	97	98	97
DenseNet169	97	97	97	97
DenseNet201	98	98	98	98
VGG16	97	97	97	97
VGG19	94	94	94	94
VGG19-FF	95	95	95	95
InceptionV3	99	99	99	99
InceptionResNetV2	99	99	99	99
AlexNet	30	14	9	30

**Table 4:** GAN-augmented dataset (300 images) model results.

Models (Batch Size 32)	Accuracy (%)	F1-Score (%)	Precision (%)	Recall (%)
Custom Model 1	97	94	95	94
Custom Model 2	87	87	87	87
DenseNet121	97	97	97	97
DenseNet169	91	91	93	91
DenseNet201	96	96	96	96
VGG16	83	83	85	83
VGG19	89	89	89	89
VGG19-FF	88	88	89	88
InceptionV3	94	94	95	94
InceptionResNetV2	90	90	90	90
AlexNet	74	75	75	74

## Data Availability

The raw data supporting the conclusions of this article will be made available by the authors on request.

## Abbreviations

The following abbreviations are used in this manuscript:

CNNs	Convolutional Neural Networks
GAN	Generative Adversarial Network
WGAN-GP	Wasserstein GAN with Gradient Penalty
FID	Fréchet Inception Distance
IS	Inception Score
FF	Fine-Tuning
ReLU	Rectified Linear Unit
TPUs	Tensor Processing Units
GPUs	Graphics Processing Units
cGAN	Conditional Generative Adversarial Network
CycleGAN	Cycle-Consistent Generative Adversarial Network
MRI	Magnetic Resonance Imaging
TP	True Positive
FP	False Positive
TN	True Negative
FN	False Negative
